# Short CRP for Anterior Canalithiasis: A New Maneuver Based on Simulation With a Biomechanical Model

**DOI:** 10.3389/fneur.2020.00857

**Published:** 2020-08-13

**Authors:** Ricardo D'Albora Rivas, Michael Teixido, Ryan M. Casserly, María Julia Mónaco

**Affiliations:** ^1^Department of ENT, Hospital de Clínicas, University of the Republic, Montevideo, Uruguay; ^2^Christiana Care Health Systems, Newark, DE, United States; ^3^Department of Otolaryngology, University of Pennsylvania, Philadelphia, PA, United States; ^4^Department of Otolaryngology, Thomas Jefferson University, Philadelphia, PA, United States; ^5^Audiology Department Arauz Otorhinolaryngology Institute (IORL Arauz), Buenos Aires, Argentina

**Keywords:** benign paroxysmal positional vertigo, anterior canalithiasis, short CRP, maneuver, biomechanical model, simulation

## Abstract

**Introduction/Objective:** Anterior canalithiasis is an uncommon and challenging diagnosis. This is due in part to the difficulty of defining the affected side, the extreme positioning required to carry out described therapeutic maneuvers, and the infrequent use of specific maneuvers. Our objective is to present a new treatment alternative for anterior canalithiasis which is based on the well-known canalith repositioning procedure (CRP) described by Epley and which is used routinely in the treatment of both posterior and anterior canalithiasis. Analysis of the standard CRP for anterior canalithiasis with a biomechanical model validates that this new maneuver is an enhanced treatment option for anterior canalithiasis. We call the new maneuver the “short CRP.”

**Methods:** A previously published 3D biomechanical model of the human labyrinths for the study of BPPV was used to analyze the conventional CRP in the treatment of anterior canalithiasis. The expected position of free otoliths near the anterior ampulla of the anterior semicircular duct was followed while recreating the sequential positions of the CRP. Although the standard CRP was possibly effective, certain enhancements were evident that could increase successful repositioning. These enhancements were incorporated into the modification of the CRP presented here as the “short CRP” for anterior canalithiasis.

**Results:** The traditional CRP used for posterior canalithiasis can also be used for anterior canalithiasis. Although in the traditional CRP the head hangs 30° below horizontal, our simulation shows that a 40° head-hang below horizontal is an enhancement and may ensure progression of anterior otolith debris. Elimination of Position 4 of the classic CRP, in which the face is turned 45° toward the floor, was also seen as an enhancement as this position is predicted to cause retrograde movement of otoliths back into the anterior canal if the patient tucks the chin in position 4 or when sitting up.

**Conclusion:** A modification of the CRP called the “short CRP” can be used to treat anterior canalithiasis. Model analysis predicts possible increased efficacy over the standard CRP. Model analysis of existing BPPV treatments is a valuable exercise for examination and can lead to realistic enhancements in patient care.

## Introduction

Anterior canalithiasis was first described in 1994 and is the least common variant of canalithiasis (1). Canalithiasis of the anterior canal produces a nystagmus with a downbeating vertical component, and with a torsional component directed toward the affected ear. In this report, Herdman et al. reported on 12% of 77 canalithiasis patients with eye movements consistent with anterior canalithiasis. The canalith repositioning procedure (CRP) had been described by Epley 2 years earlier and was used successfully in these patients with anterior canalithiasis (2). The CRP has remained in the toolbox as a primary treatment for anterior canalithiasis ever since. Subsequent systematic literature review has established the prevalence of anterior canalithiasis at 3% of cases of BPPV (3).

Later investigators have explored many other ways to effect repositioning of debris in the distal anterior canal back into the utricle. In 1999, a reverse Epley maneuver was described in which the head is dropped into the Dix-Hallpike position with the affected ear up and the patient is then moved in 90° steps toward the unaffected side as in the CRP (4). In 2004, another variation was described which can be accomplished simply with side-lying onto the affected side with the head hanging 45° below horizontal, then rising in steps to horizontal and then to 45° above horizontal before sitting up (5). In 2004, the Prolonged Forced Position Procedure was introduced (6). Although it was an impractical, hours long inpatient treatment—making it too cumbersome for practical use—the technique proved that extreme head hanging in the midline with sequential rising to upright could be effective regardless of the side affected. Other investigators showed that rising to upright in much shorter intervals of only 1 min from the Dix-Hallpike to the unaffected side and the affected side was effective (7). Subsequently, when rising at these intervals the Dix-Hallpike position on the affected side was also found to be effective (8, 9). Finally the advantages of midline head hanging without regard to the affected side and with faster sequential rising to sitting were combined by Yacovino who showed success starting with the head hanging 30–45° and rising to 45° above horizontal for 30 s before rising to sitting (10). This Yacovino maneuver has remained, like the CRP, a part of the common treatment canon for anterior canalithiasis. Yacovino's maneuver was subsequently re-described with subtle differences: a 3 min pause in each position rather than 30 s, and rapid transitions (11).

Today, there is no consensus on the best treatment for anterior canalithiasis. The Yacovino maneuver and the CRP are perhaps the most familiar to most practitioners. Efficacy of various repositioning strategies for anterior canalithiasis is only 75% (3). This is lower than efficacy reported for posterior canalithiasis treatment (1, 8, 12). In this study, we performed analysis of the CRP as used for anterior canalithiasis using a biomechanical model and identified a simplification that may result in improved efficacy (13). This simplified maneuver is presented here and called the “short CRP.”

## Materials and Methods

A 3D model developed for the study of otolith disease was used to visualize the treatment of anterior canalithiasis by studying expected otolith positions in the different phases of the CRP maneuver. Our 3D model of the human membranous labyrinth, as previously reported, was created following the same technique as reported by Teixido et al. and Wang et al. for the creation of the Download-able Virtual Model of the Temporal Bone (13, 14). The model was created from axial histological sections, which were imaged with high resolution scanning and integrated into Amira 5.2.2. The reconstructed labyrinth was cloned for the contralateral side and carefully positioned in relation to the 3D surface map of a human skull and then a skin surface was applied. Moveable markers for otoconia were created to allow known and expected positions of otoconia to be mapped while transitioning from position to position.

As the head was moved into different positions during the CRP for anterior canalithiasis, the new gravity-dependent position of the otolith mass was marked. The standard CRP maneuver sequence was followed with an otolith mass present in the right anterior canal. The classic sequence was modified to maximize forward progression, and to avoid unnecessary positions and retrograde movement of the otolith mass during repositioning. Numerous trials resulted in identification of a modified sequence which maximizes progression and reduces retrograde movement of the otolith mass. Screenshots were taken for the publication of this article.

## Results

**Figure 1:**
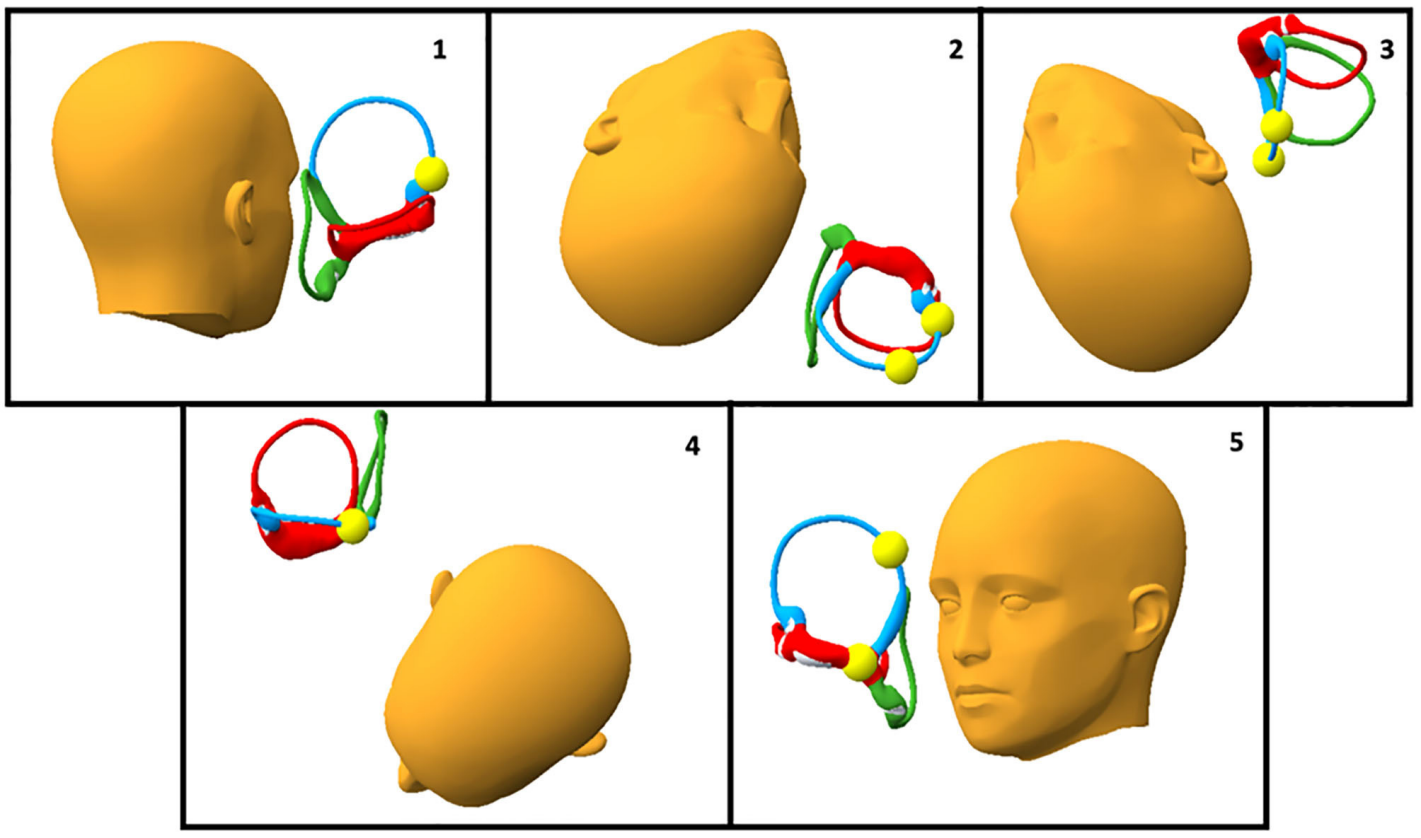
The classic CRP has five positions shown here in a case of right anterior canalithiasis: In Position 1 the patient is seated upright with the head turned 45° to the affected side. In Position 2 the head hangs 30° below horizontal while turned 45° to the right. Position 3 is shown with the head hanging 30° and the head turned 45° to the left. Position 4 is shown with the patient rolled onto the left shoulder and with the face turned 45° toward the floor. In Position 5 the patient returns to sitting upright. Expected progression of the otolith mass is shown.

**Figure 2:**
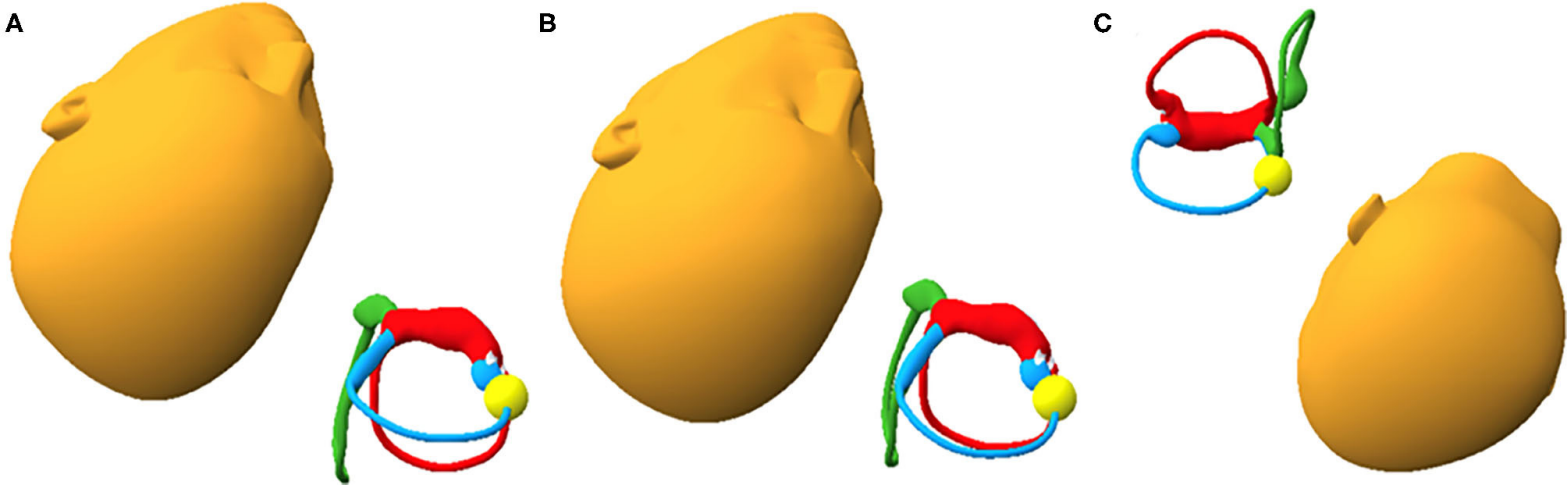
**(A)** Shows anterior canalith position (yellow sphere) on the right with the head hanging 30° below horizontal and the head turned 45° to the right. **(B)** Shows anterior canaliths on the right with the head hanging 40° below horizontal and the head turned 45° to the right. Otolith movement is likely enhanced with greater head-hang. **(C)** The effect of tipping the head forward in position 4. In this circumstance otoliths may move back into the anterior semicircular duct and are in danger of resuming their starting position if the chin is tucked on rising.

Our analysis demonstrated the reported efficacy of the CRP for treatment of anterior canalithiasis with progression of otolith debris around the circumference of the anterior canal during the CRP (Figure 1). It also revealed potential enhancements and possible pitfalls of the traditional Epley for treating anterior canalithiasis that can influence the effectiveness of the maneuver for anterior canalithiasis that are not obvious without model analysis. An enhancement is hanging the head to lower than 30° in position 2 to promote more definite progression of the otolith mass around the circumference of the anterior canal (Figure 1, Position 2). Figures 2A,B demonstrate the head hanging 30° and 40° below horizontal. The potential benefit of greater head hang than usual in the CRP is evident.

The most notable potential pitfall of the CRP is the position of the chin in head position 4. As seen in Figure 1, in Position 4 the chin is not tucked and the anterior canal is parallel to the earth so no otolith movement is expected. If the chin is tucked, however, as in Figure 2C, the otolith mass can progress in a retrograde fashion into the anterior canal. Sitting up with the chin tucked from this position could result in the return of otoliths to their starting position and a treatment failure.

**Figure 3:**
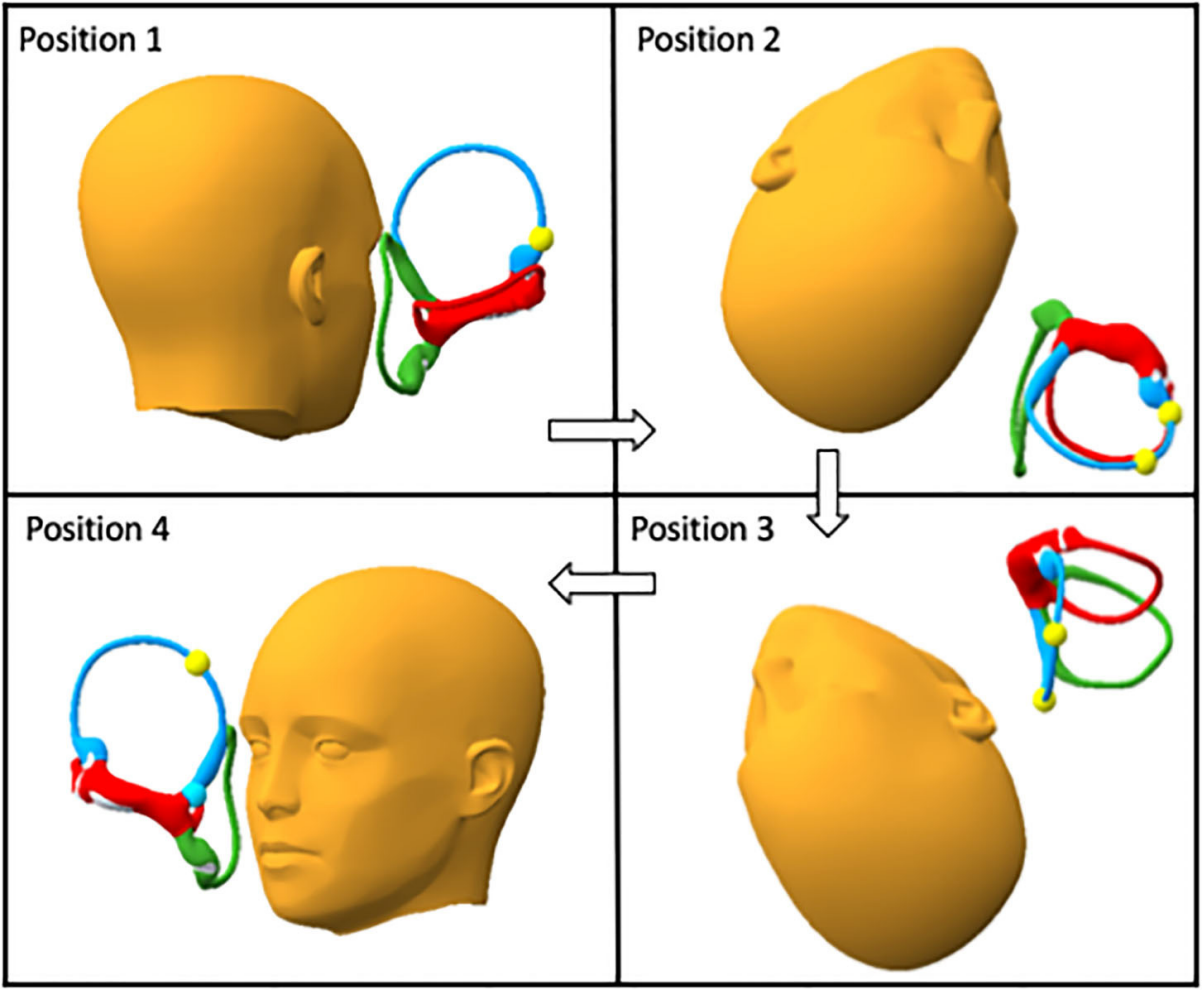
The short Epley for anterior canalithiasis has four positions shown here: In Position 1 the patient is seated upright with the head turned 45° to the affected side. In Position 2 the head hangs 40° below horizontal while turned 45° to the right. Position 3 is shown with the head hanging 40° and the head turned 45° to the left. In Position 4 the patient returns to sitting upright. Expected gravitationally motivated progress of the otolith mass is shown as yellow spheres which mark positions before and after each position.

Evident from this analysis is that Position 4 of the CRP may be omitted altogether, avoiding a potential pitfall and simplifying the maneuver. The shortened maneuver with increased head hang is presented here as the “short CRP” in Figure 3.

## Discussion

The treatment of canalithiasis has been characterized by constant modification and refinement. A review of the history of treatment of anterior canalithiasis presented above demonstrates that attempts at modification often serve only to prove another unique way to accomplish the same goal of particle repositioning. These can have their useful place if they serve the needs of selected patients with mobility and positioning problems. In our experience the maneuvers most utilized in the treatment of anterior canalithiasis are the Yacovino and the CRP. These have found their place in treatment based on their utility in the case of the Yacovino which does not require identification of the affected side, and familiarity in the case of the CRP. Both maneuvers are effective. Our analysis of the CRP in anterior canalithiasis presented in this paper is an attempt to provide a refinement that can enhance current therapy of patients with anterior canalithiasis who are currently treated with CRP.

Anterior canalithiasis treatment has been poorly studied and treatment efficacy is lower than treatment for posterior canalithiasis (12). This may be due to the difficulty in identifying rare patients for case series study, or because of the difficulties inherent in the diagnosis of anterior canal disease. These difficulties may include challenges in identifying the affected side because of an imperceptible rotary component of nystagmus. Since the position of the anterior canal axis on the globe is nearly equatorial, the rotary component is not as evident as in posterior canal disease. In some patients, downbeat nystagmus may be masked by concurrent posterior canal disease provoked in the same Dix-Hallpike position. Additionally, a patient thought to have anterior canalithiasis may actually have apogeotropic posterior canalithiasis or common crus lithiasis that escapes the attention of the examiner. The separation of these entities which may cause downbeating nystagmus from anterior canalithiasis is a subject of ongoing discussion (15). Other challenges to accurate diagnosis exist. Some central positional downbeat nystagmus may be incorrectly diagnosed as BPPV. Treatment deficiency may also be due to unrecognized errors in performance of maneuvers created by difficulties the practitioner may have in visualizing the anterior canal and the membranous labyrinth in general. The ability to clearly visualize the labyrinth is possible if an accurate model is utilized. It is from this perspective that our re-analysis of existing treatments is oriented.

It is reasonable to question the utility of model analysis in BPPV treatment. The authors acknowledge that although the model is based on a human membranous labyrinth the model is based on only a single labyrinth. It resides within the bony labyrinth which itself has small but significant variations of position within the human skull (16). As such, the model may not be said to be a final predictor of all possible otolith movement phenomena related to BPPV. Other sources of variable otolith behavior such as otolith size and proximity to the duct wall have been proposed in empiric study (17). These proposed variables as well as other known phenomenon of otolith movement such as canal conversion and canalith jam may also confound model predictions. Our model comprises a freely mobile head whose positioning is not constrained by a neck and body and we have taken care to avoid positioning that is anatomically impossible. The modifications proposed are within the well-established range of movements required in the standard CRP. We feel it is reasonable to trust model analysis if the predicted otolith movements are gross movements and are reasonably similar to head position changes that produce observable eye movements in clinical practice and in maneuvers with validated efficacy as in posterior canalithiasis. A biomechanical analysis of the Dix -Hallpike maneuver was previously reported which resulted in the introduction of an expanded Dix-Hallpike maneuver which has added clinical utility by allowing separation of posterior and anterior canal responses in patients who may have simultaneous disease (18).

Our proposed maneuver has some disadvantages over the commonly used Yacovino maneuver in that it requires determination of the affected side, which can be difficult in anterior canalithiasis, and because it has more head positions than the Yacovino. Our hope is that some patients found to have anterior canalithiasis who cannot extend their necks sufficiently in the midline supine position may be effectively treated with this adaptation of the CRP.

Our current analysis has resulted in a simplification and enhancement of the CRP when used for anterior canalithiasis. The simplification eliminates the unnecessary Position 4 in the CRP treatment sequence which may compromise efficacy, and the enhancement includes head hanging below 30° to more definitely facilitate otolith progression in a direction that promotes maneuver success. We believe the “short CRP,” comprised of modifications of the well-known CRP, may be an option to treat anterior canalithiasis. Successful performance on human subjects is required to prove its efficacy we believe the “short CRP,” with these resulting modifications of the well-known CRP, can be used to treat anterior canalithiasis.

## Conclusion

A modification of the CRP called the “short CRP” may be an option to treat anterior canalithiasis. Model analysis demonstrates possible increased efficacy over the standard CRP. Model analysis is a valuable exercise for examination of existing BPPV treatments and can lead to realistic enhancements in patient care.

## Data Availability Statement

The original contributions presented in the study are included in the article/supplementary material, further inquiries can be directed to the corresponding author/s.

## Author Contributions

RD'A: original concept. MT: biomechanical analysis, manuscript creation, and illustrations. RC: manuscript preparation. MM: original concept. All authors contributed to the article and approved the submitted version.

## Conflict of Interest

The authors declare that the research was conducted in the absence of any commercial or financial relationships that could be construed as a potential conflict of interest.
